# Cross-species mapping of acute psychedelic-responsive genes links HTR2A cortical expression, cell-type enrichment, and human cortical expression modules

**DOI:** 10.1038/s41398-026-04349-z

**Published:** 2026-08-01

**Authors:** Patrick A. McConnell, Jackson Raffety, Andrew Li, Eric Steinberg, Syed Rahim, Christian Valtierra, Adam Gazzaley, Robin L. Carhart-Harris, Lorenzo Pasquini

**Affiliations:** 1 Department of Psychiatry and Behavioral Sciences, University of California, San Francisco, CA USA; 2 Department of Neurology, University of California, San Francisco, CA USA

**Keywords:** Molecular neuroscience, Human behaviour

## Abstract

Psychedelic drugs exert rapid and profound effects on human consciousness [1–7] and are increasingly being investigated for their therapeutic potential [8–14]. Yet the transcriptional programs through which they may reshape brain function and structure remain incompletely understood, in part because the available evidence is heterogeneous and largely preclinical [15, 16]. In a structured scoping review of transcriptomic studies in animal models and neural cell cultures, we identified genes reported in the source literature to change within 3 h of classical psychedelic administration and intersected them with the Allen Human Brain Atlas to define an acute expression set. This retained set showed cortex-centered enrichment, a marker-based deep-layer projection-neuron component, and ontology enrichment for neuron projection and related synaptic terms. A smaller subset overlapped with human accelerated region-associated genes and showed greater-than-random spatial alignment with a human cortical-expansion map. The retained cortical-expression maps showed serotonergic spatial alignment in the human cortex, most prominently with *HTR2A* expression. Clustering identified three cortical spatial-expression modules, including an association/limbic-weighted module enriched for plasticity and synaptic-organization terms. Together, these findings provide a human cortical framework linking acute psychedelic-responsive transcription to cortical cell-type enrichment, *HTR2A* spatial expression, cortical spatial-expression modules, and cortical-expansion, while leaving receptor-specific, causal, translational, and cell-type-resolved mechanisms for future work.

## Introduction

Serotonergic psychedelics such as psilocybin, lysergic acid diethylamide (LSD), and N,N-dimethyltryptamine (DMT) have re-emerged as compounds of major interest in psychiatry and neuroscience due to their profound effects on the brain affecting perception, mood, and cognition [1–6]. Multiple clinical trials now report rapid and, in some cases, sustained symptom improvement across several clinical populations [8–14].

A growing literature suggests that psychedelics engage neuroplasticity-related processes in the mammalian brain, including dendritic growth, synaptogenesis, and transcriptional reprogramming [17–21]. Transcriptomic studies and reviews in animal models have also reported widespread changes in gene expression following psychedelic administration, implicating molecular processes relevant to neuronal signaling and plasticity [15, 16, 21]. Despite recent advances in relating psychedelic-associated functional and molecular signatures to human neurotransmitter and transcriptomic maps [22–24], it remains unclear how these preclinical transcriptional signatures relate to the human brain, which provides the biological substrate for the therapeutic and experiential effects of psychedelics.

At the receptor level, classic serotonergic psychedelics act principally through the serotonin 2A (5-HT2A) receptor, encoded by *HTR2A* [7]. In the human cortex, 5-HT2A receptor architecture is especially prominent in higher-order association territories [25]. Postmortem work also shows structured *HTR2A* expression in human prefrontal cortex [26]. These higher-order cortical areas have been repeatedly implicated in psychedelic-related phenomenology and large-scale functional reorganization [1, 3, 6, 23, 27–29]. Many of these brain areas have also undergone marked evolutionary expansion in humans [30, 31], with studies suggesting that human-accelerated regions (HAR)—short stretches of non-coding DNA showing rapid evolutionary change in humans—are preferentially expressed in cortically expanded areas overlapping with brain regions expressing the 5-HT2A receptor [25, 26, 30–33]. These converging findings suggest that acute psychedelic-responsive transcriptional changes may map onto human cortical systems linked to higher cognition and recent cortical evolution.

To address this question, we combine gene-expression data from preclinical psychedelic studies with the Allen Human Brain Atlas (AHBA) and evolution-linked gene annotations. Because this literature remains heterogeneous and preclinical, we first use a structured scoping review to define an acute gene set changing expression within 3 h of psychedelic administration and then map that set into the human brain. We test whether that set shows cortex-centered enrichment and spatial correspondence with cortical expression maps of serotonergic and dopaminergic markers. We also test whether it forms separable cortical spatial-expression modules, overlaps with HAR-associated genes, and shows exploratory correspondence with global functional connectivity from a published human LSD fMRI dataset [1]. This framework allows us to evaluate how a retained acute transcriptional signal maps onto human cortical organization.

## Methods

### Literature search of neural-expression changes after psychedelic administration

Between January 1 and May 30, 2025, we conducted a PRISMA-informed scoping review [34] of PubMed and Google Scholar for primary peer-reviewed studies reporting neural gene-expression changes after administration of a classic serotonergic psychedelic. Records were imported into Covidence for deduplication and title/abstract screening [35]; detailed search strings, screening procedures, and extraction rules are provided in Supplementary Appendix S1. Eligibility was defined a priori with a PICO-S framework [36]. We retained peer-reviewed primary studies that reported or allowed recovery of gene-level neural-expression changes from brain tissue or neural cell culture under an eligible control.

Genes were included in the retained “genes reported to change expression acutely under psychedelics” (GCEP) set if they were reported at sampling times within 3 h of dosing, including the 3 h timepoint [21, 37–50]. We used the within-3 h boundary to retain the empirically dense early post-dose interval. Genes overlapping with the AHBA background gene pool were retained for downstream human spatial analyses (GCEP + AHBA). AHBA overlap denoted gene-level availability in the AHBA expression background; cortical restriction was applied downstream when the retained genes were mapped onto Schaefer-200 cortical parcels. Observations outside the retained within-3 h window were preserved for descriptive timing and scope summaries.

A study event was defined as a unique combination of PMID, drug, species, sex, exact reported post-dose sampling time, and evaluation duration when separately reported; sampled region was not part of this key. Gene labels were harmonized to the symbol-resolved AHBA namespace. Ambiguous or non-one-to-one cross-species mappings were not imputed. Retention for downstream human spatial analyses required overlap with the AHBA background gene pool rather than source-study cortical sampling alone. Thus, retained genes observed in noncortical source-study samples could still enter downstream cortical Schaefer-space maps when their expression was available in AHBA.

### Human-expressed genes from the Allen Human Brain Atlas

Human gene-expression data used for cortical mapping were derived from AHBA microarray data [51]. The dataset includes 3702 tissue samples from six adult donors, with bilateral sampling in two donors and left-hemisphere sampling in four donors, and provides expression estimates for 20,734 genes across 58,692 probes [51]. We processed these data with abagen [52] by updating probe annotations, filtering probes that did not exceed background, selecting one representative probe per gene, assigning samples to the cortical Schaefer-200 atlas [53], normalizing expression values, removing genes inconsistently expressed across donors, and averaging across donors. This yielded a Schaefer-200 cortical expression matrix with 15,633 symbol-resolved genes; 11 parcels lacked expression values across all genes, leaving usable cortical expression estimates for 189 of 200 cortical parcels (Supplementary Figure S2; exact parcel list in Supplementary Appendix S2). The same 15,633-gene symbol-resolved cortical expression background was used for overlap analyses and permutation-based testing (see more details below). Although the AHBA includes both cortical and subcortical samples, the present analyses were restricted to the cortex due to substantial differences in gene expression values when comparing subcortical and cortical parcels. We therefore did not jointly analyze cortical and subcortical bulk-expression distributions without a separate harmonization workflow [52, 54]. Additional processing details are provided in Supplementary Appendix S2.

### Gene ontology and cell enrichment analyses

The retained acute psychedelic-responsive AHBA-overlap set (GCEP + AHBA; n = 51) was entered into a selective enrichment workflow. This included Gene Ontology Cellular Component and Biological Process libraries via Enrichr [55] and SynGO [56]. The Dougherty reference resource [57] was used for cell-type-specific enrichment analysis (CSEA) and for complementary specific enrichment analysis (SEA) of adult regions and developmental stages. These enrichment families were interpreted within the native reference terms supplied by Enrichr, SynGO, and the Dougherty CSEA/SEA resource rather than against a separately remapped 15,633-gene AHBA cortical background, which was reserved for overlap and permutation testing. Benjamini-Hochberg-corrected significance metrics were used for the main enrichment summaries.

### Cortical spatial correlations with HTR2A and comparator marker maps

Regional cortical AHBA-derived gene-expression maps for *HTR1A, HTR2A, HTR2C, DRD1*, and *SLC6A4* were derived from the same AHBA-to-Schaefer-200 processing pipeline described above. Of the comparator marker genes, only *HTR2A* was part of GCEP + AHBA; therefore, *HTR2A* was removed before all marker-map alignment analyses, yielding a self-excluded 50-gene set. For each retained gene, we computed Pearson correlations between its cortical expression map and each receptor map.

To test whether this self-excluded 50-gene set aligned with the cortical *HTR2A* expression map more strongly than expected by chance, we generated a null distribution of mean *HTR2A*-map correlations from 5000 randomly sampled same-size non-GCEP, non-*HTR2A* gene sets derived from the AHBA gene pool. For each permutation, we calculated the average correlation between the sampled random gene-expression maps and the *HTR2A* cortical expression map. The empirical *p*-value was defined as the proportion of null means equal to or greater than the observed average correlation for the self-excluded GCEP + AHBA set.

One fixed same-size AHBA comparison set was selected for visualization purposes, with nominal significance reported using a two-sided Welch’s *t*-test. For the broader receptor marker panel, the self-excluded GCEP + AHBA set was also correlated with the other receptor cortical gene-expression maps. Correlation differences were assessed with a repeated-measures ANOVA using gene as the blocking factor, followed by Benjamini-Hochberg-corrected paired contrasts across the full marker panel. Exact pairwise *t* statistics, adjusted *p*-values, paired effect sizes (d_z_), and omnibus η^2^ are reported for supported contrasts.

### Cortical co-expression network and spatial-expression module analysis

To test whether the retained acute set separated into distinct cortical spatial-expression motifs, we constructed a gene-by-gene spatial co-expression matrix by correlating Schaefer-200 cortical expression profiles across parcels for all genes in GCEP + AHBA. For the graph analysis, we thresholded the matrix at Pearson *r* ≥ 0.3 to retain positive edges only, and binary degree was retained as a descriptive connectedness measure. We then applied the Louvain community detection from MATLAB’s Brain Connectivity Toolbox [58], using a fixed random seed of 1 and a resolution parameter of 0.5. Gene identities within each detected community were used to generate average cortical expression maps for each cluster.

For display and canonical-network summaries, each cluster map was converted to within-cluster parcel z-scores across finite represented Schaefer parcels, so values above or below zero indicate parcels above or below that cluster’s mean cortical expression. Cluster-average cortical-expression maps were summarized across the seven canonical Schaefer networks: Visual Network (VN), Somatomotor Network (SMN), Dorsal Attention Network (DAN), Ventral Attention Network (VAN), Limbic Network (LiN), Frontoparietal Network (FPN), and Default Mode Network (DMN).

For between-network contrasts, each cluster map was evaluated with a conventional parcel-level one-way ANOVA across the seven canonical networks, with Benjamini-Hochberg correction applied across the three cluster-level omnibus tests. For clusters with a corrected significant omnibus result, post hoc network contrasts were evaluated with Welch *t*-tests and Benjamini-Hochberg correction within each cluster-specific pairwise family. Separately, within-network centering relative to the cluster mean was evaluated with two-sided one-sample *t*-tests against zero for each cluster-network parcel distribution, with Benjamini-Hochberg correction across all 21 cluster-network tests; significant effects were labeled as above or below the cluster’s cortical mean based on the observed network mean.

### Human accelerated region overlap and cortical-expansion analysis

We next tested whether the GCEP + AHBA set contained more HAR-associated genes than would be expected by chance. HAR-associated genes were identified from a comparative genomic analysis that reported 2737 HAR loci corresponding to 2164 unique HAR-associated genes [32]. Intersecting this HAR-associated gene list with the AHBA cortical-expression background yielded 1372 HAR-associated AHBA-overlapping genes (HAR + AHBA) [32, 51]. Overlap between HAR + AHBA (n = 1,372) and the GCEP + AHBA set (n = 51) was assessed with a one-tailed Fisher exact test in R [59].

For the narrowed HAR-overlap subset (HAR + GCEP + AHBA; n = 9), mean cortical expression was visualized in Schaefer-200 space and summarized across the seven canonical Schaefer networks [52–54]; the network view and its conventional parcel-level omnibus ANOVA were retained as descriptive context only. We then compared the nine HAR-overlap gene-expression maps with the cortical-expansion map from Wei et al. [30] in matched Schaefer-200 space, using the mean gene-wise spatial correlation as the observed statistic. The observed mean spatial correlation was tested against a null distribution generated from 5000 same-size gene sets sampled without replacement from the AHBA cortical-expression background after excluding the observed HAR-overlap genes, with the empirical one-sided *p*-value defined as the proportion of null means greater than or equal to the observed mean. The HAR-overlap cortical maps and Wei et al. cortical-expansion map were evaluated as matched Schaefer-200 cortical parcel vectors rather than as an image-space overlap test [30, 51–54].

### Bounded psychostimulant comparator analyses

As a secondary context analysis, we compared the GCEP + AHBA set with two acute psychostimulant comparator sources selected outside the primary PICO-S-bounded psychedelic review; source selection, reconstruction, and mapping procedures are summarized in Supplementary Appendix S1.

For cocaine, we used the acute prefrontal-cortex differential-expression source from Emerson et al. [60], corresponding to the prefrontal cortex (PFC) Saline-Cocaine vs Saline-Saline comparison in Extended Data 3. We retained two mapped comparison views: a broader nominal *p* < 0.05 list with 352 source rows and 343 mapped current symbols, and a stricter nominal *p* < 0.01 sensitivity list with 56 source rows and 52 mapped current symbols. Because the source used mouse Ensembl-style identifiers, overlap was assessed after Ensembl-to-symbol mapping. Unresolved or archived identifiers were excluded from direct matching.

For amphetamine, we used the day-1 dorsal-striatal d-amphetamine source from Crittenden et al. [61], reflecting tissue harvested 20 min after the first injection, and treated the dorsal-striatal source tissue as an explicit interpretative limit. The primary amphetamine comparison used a reconstructed 44-gene acute list; broader 61- and 62-gene support views were retained only as sensitivity context.

In both comparator analyses, overlap with the psychedelic set was counted by exact symbol matching against the fixed 51-gene GCEP + AHBA set.

### Exploratory psychedelic functional-topography comparison

We used a preserved parcel-level LSD-minus-placebo resting-state global functional connectivity (GFC) vector in Schaefer-200 space [1]. Spatial similarity between the LSD-minus-placebo GFC map and each gene-cluster expression map was assessed across the same 189 AHBA-complete Schaefer parcels used for the cluster maps using Pearson correlation. Empirical spatial-null *p*-values were estimated from 5000 BrainSMASH surrogate maps that preserved the autocorrelation structure of the LSD-minus-placebo GFC map [62]. Tail probability was counted one-sided in the observed direction of effect for each cluster. Benjamini-Hochberg correction was applied across the three cluster-versus-GFC tests.

## Results

### Evidence base and retained acute GCEP + AHBA set

**Fig. 1:**
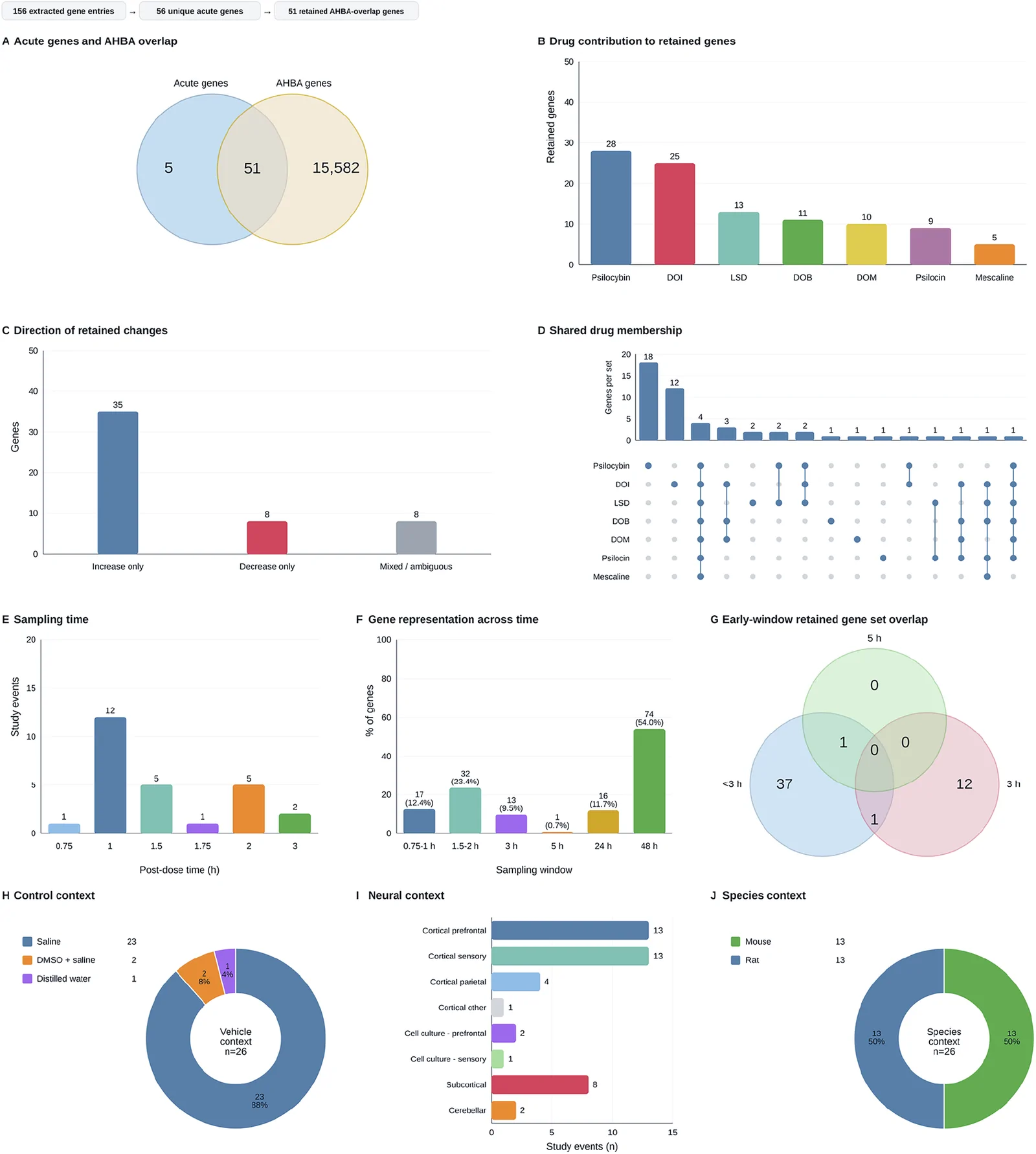
Scoped evidence base and retained acute psychedelic-responsive AHBA-overlap gene set. The top summary strip shows set construction across the scoped corpus: 156 extracted gene entries, 56 unique genes reported within 3 h of dosing, and 51 retained AHBA-overlap genes (GCEP + AHBA). Panels **A**-**D** summarize retained-gene construction and membership patterns, including the GCEP/AHBA overlap defining the retained set (acute-only n = 5; AHBA-only n = 15,582; overlap n = 51), drug contribution, expression-change direction, and shared drug membership. Panels **E**-**J** summarize timing and study-event context, including sampling-time distribution, collapsed time-bin representation, early-window overlap across <3 h/exact 3 h/exact 5 h bins, control condition, sampled neural context, and species. AHBA: Allen Human Brain Atlas; DMSO: dimethyl sulfoxide; DOB: dimethoxybromoamphetamine; DOI: 2,5-dimethoxy-4-iodoamphetamine; DOM: dimethoxymethylamphetamine; GCEP: genes reported to change expression acutely under psychedelics; LSD: lysergic acid diethylamide.

Our scoping review search (N = 474) yielded 324 unique articles for title/abstract screening and 86 records for full review (Fig. 1A–J). After nine initial full-text exclusions and a manual eligibility review, two additional qualifying study records were added, yielding 79 unique study records. These yielded 17 eligible studies meeting our PICO-S criteria and reporting neural gene-expression changes after classic serotonergic psychedelic administration (Supplementary Figure S1; Supplementary Table S1; Supplementary Appendix S1).

We extracted metadata for 156 gene entries (Supplementary Table S4). Fifty-six unique genes were reported at sampling times within 3 h of dosing (mean = 1.46 h; SD = 0.61 h; range = 0.75–3 h) (Fig. 1E–G; Supplementary Table S4). Of these 56 genes, 51 overlapped the AHBA background gene pool, defining the retained acute AHBA-overlap set (GCEP + AHBA; Fig. 1A). Within GCEP + AHBA, the mixed/ambiguous directional accounting comprised *ARC*, *BDNF*, *EGR2*, *EGR3*, *LYN*, and *STYK1*, whereas *NEGR1* and *NPTX1* were retained without a clear signed acute-response direction (Fig. 1D). Drug-membership overlap was otherwise sparse, with *EGR1*, *EGR2*, *FOSB*, and *NFKBIA* the only retained genes shared across all represented drug categories (Fig. 1D).

The retained within-3 h evidence base comprised 26 study events across 15 PMIDs; 23/26 were sampled between 1 and 2 h and 23/26 used saline controls. A stricter <3 h boundary would have excluded 12 AHBA-overlapping genes represented only at exactly 3 h, whereas expanding the window to ≤12 h or adding exact 5 h observations did not add AHBA-overlapping genes. Later AHBA-overlap observations were limited to a 24 h psilocybin pig study [63] and an LSD-only >24 h mouse lineage and were retained for descriptive scope only (Supplementary Figure S3; Supplementary Table S3).

### Gene enrichment and ontology of the retained acute set

**Fig. 2:**
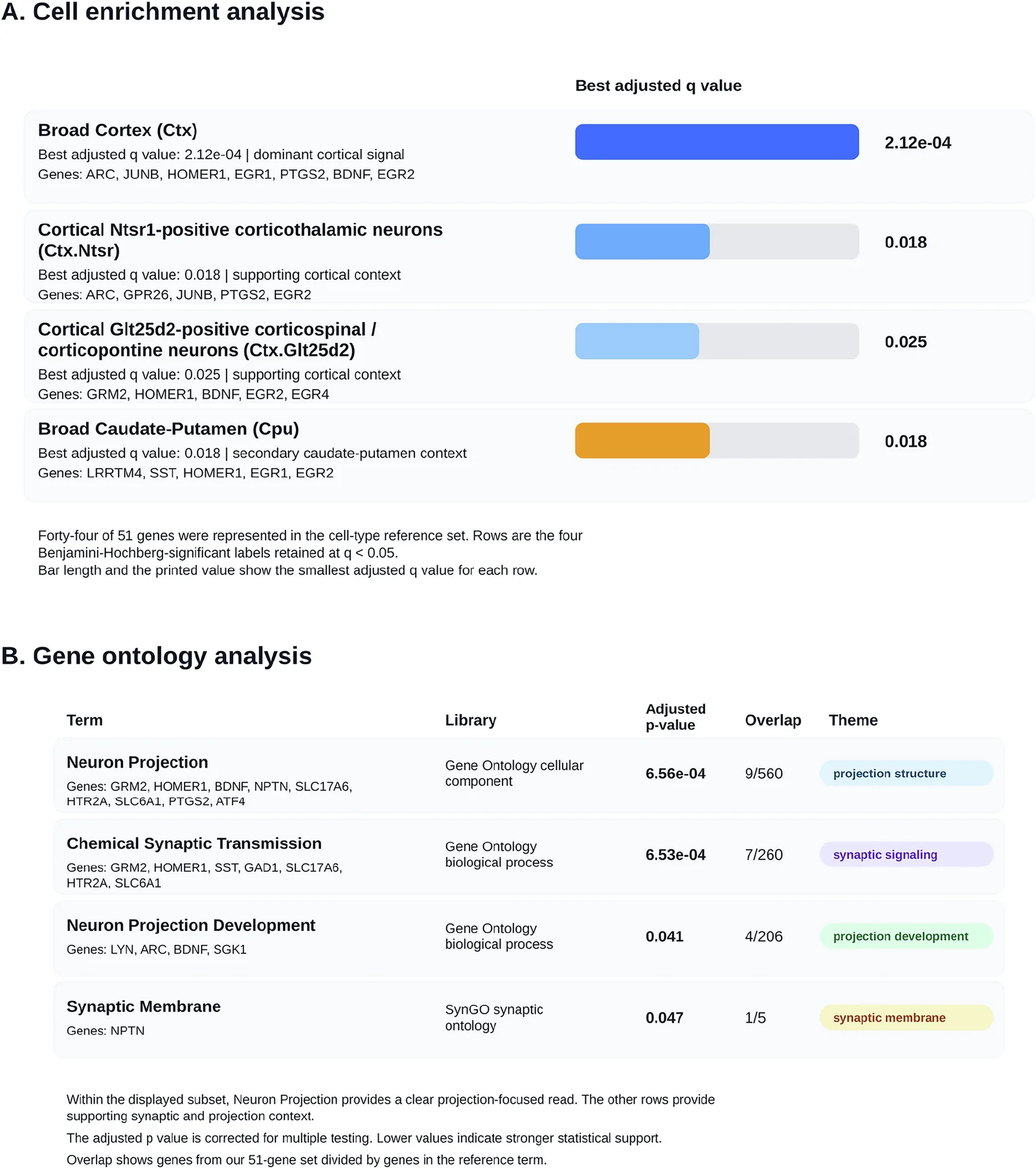
Cortex-centered enrichment of the retained acute psychedelic-responsive GCEP + AHBA gene set. Panels A-B summarize displayed CSEA and ontology results for the retained GCEP + AHBA set (n = 51): cell-type-specific enrichment with representative overlapping genes for each displayed row (**A**), and selective Gene Ontology Cellular Component, Gene Ontology Biological Process, and SynGO terms (**B**). GCEP: genes reported to change expression acutely under psychedelics; AHBA: Allen Human Brain Atlas; Ctx: cortex; Cpu: caudate-putamen; SynGO: synaptic gene ontology.

Cell-type enrichment (CSEA), ontology enrichment, and complementary SEA supported a cortex-centered enrichment pattern in the GCEP + AHBA set with 44 of 51 genes represented in the CSEA/Dougherty reference. Four CSEA labels survived *q* < 0.05: broad cortex (Ctx; *q* = 2.12 × 10^−4^), broad caudate-putamen (Cpu; *q* = 0.02), cortical Ntsr1-positive corticothalamic neurons (Ctx.Ntsr; *q* = 0.02), and cortical Glt25d2-positive corticospinal/corticopontine neurons (Ctx.Glt25d2; *q* = 0.03) (Fig. 2A). Complementary SEA showed strong enrichment for Cortex.Young.Adulthood (*q* = 7.99 × 10^−7^) and additional cortical developmental terms spanning neonatal/early infancy through adolescence.

The Broad Cortex category lacked row-specific genes and was driven by recurring activity- and plasticity-linked genes (*ARC, JUNB, HOMER1, EGR1, PTGS2, BDNF*, and *EGR2*) shared across rows. Narrower cortical rows introduced row-specific signals, with *GPR26* for Ctx.Ntsr and *GRM2/EGR4* for Ctx.Glt25d2, highlighting a deep-layer projection-neuron motif within the same cortex-centered pattern. In contrast, the secondary striatal Cpu category added *LRRTM4* and *SST*. Ontology summaries complemented the cortex-centered CSEA/SEA pattern by identifying neuron-projection and synaptic-organization terms in the same retained gene set (Fig. 2B).

Among displayed terms surviving adjusted *p* < 0.05, “Neuron Projection” added *ATF4* and *PTGS2*, “Chemical Synaptic Transmission” added *GAD1* and *SST*, “Neuron Projection Development” added *ARC, LYN*, and *SGK1*, and “Synaptic Membrane” contributed *NPTN*. Overall, Fig. 2, supports a cortex-centered enrichment pattern, alongside secondary striatal and developmental signals.

### Serotonergic spatial alignment in human cortex

**Fig. 3:**
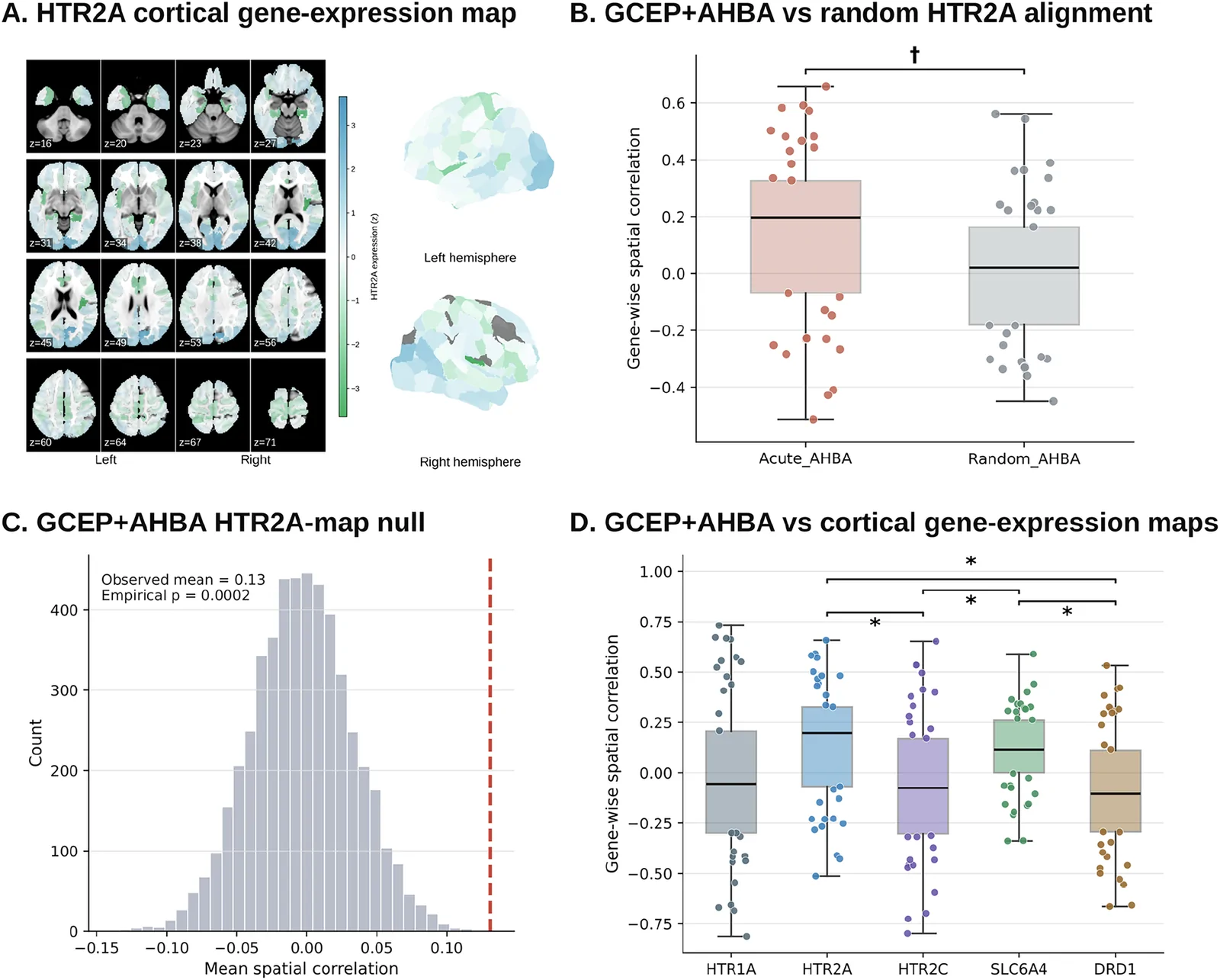
Serotonergic spatial alignment of the retained acute psychedelic-responsive GCEP + AHBA gene set in human cortex. Analyses used the self-excluded retained subset (GCEP + AHBA excluding *HTR2A*; n = 50) and AHBA-derived cortical maps for *HTR1A, HTR2A, HTR2C, SLC6A4*, and *DRD1*. Panel **A** shows the AHBA *HTR2A* cortical expression map; panel **B** shows one fixed same-size AHBA comparison set for visual context only, with the dagger marking the nominal displayed contrast (two-sided Welch *p* = 0.03); panel **C** shows the primary 5000-resample *HTR2A*-map null; and panel **D** shows secondary gene-wise marker-context comparisons, with asterisks marking Benjamini-Hochberg-adjusted paired contrasts with *p* < 0.05. GCEP: genes reported to change expression acutely under psychedelics; AHBA: Allen Human Brain Atlas.

We next asked whether the cortical gene-expression pattern of GCEP + AHBA (excluding *HTR2A*) showed above-chance alignment with the cortical *HTR2A* expression map, and whether that relationship was specific to *HTR2A* or better interpreted within a broader serotonergic and/or dopaminergic cortical expression pattern (Fig. 3).

Figure 3B, shows one same-size non-GCEP + AHBA, non-*HTR2A* AHBA comparison set for visual context (mean *r* = 0.02 versus 0.13 for the self-excluded GCEP + AHBA set; two-sided Welch *t*(95.02) = 2.14, *p* = 0.03, *d* = 0.43). The corresponding 5000-resample null showed that the observed average *HTR2A*-map correlation for the self-excluded GCEP + AHBA set remained above chance (mean *r* = 0.13; empirical *p* = 2.0 × 10^−4^) (Fig. 3C).

Across the broader cortical gene-expression panel (Fig. 3D), the within-gene omnibus test was also significant (*F*(4,196) = 5.48, *p* = 3.32 × 10^−4^, η2 = 0.07). Benjamini-Hochberg-corrected paired contrasts supported *HTR2A* > *HTR2C* (*t* = 2.51, adjusted *p* = 0.04, d_z_ = 0.36), *HTR2A* > *DRD1* (*t* = 2.80, adjusted *p* = 0.04, d_z_ = 0.40), *SLC6A4* > *HTR2C* (*t* = 2.64, adjusted *p* = 0.04, d_z_ = 0.37), and *SLC6A4* > *DRD1* (*t* = 2.88, adjusted *p* = 0.04, d_z_ = 0.41).

### Cortical spatial-expression modules

**Fig. 4:**
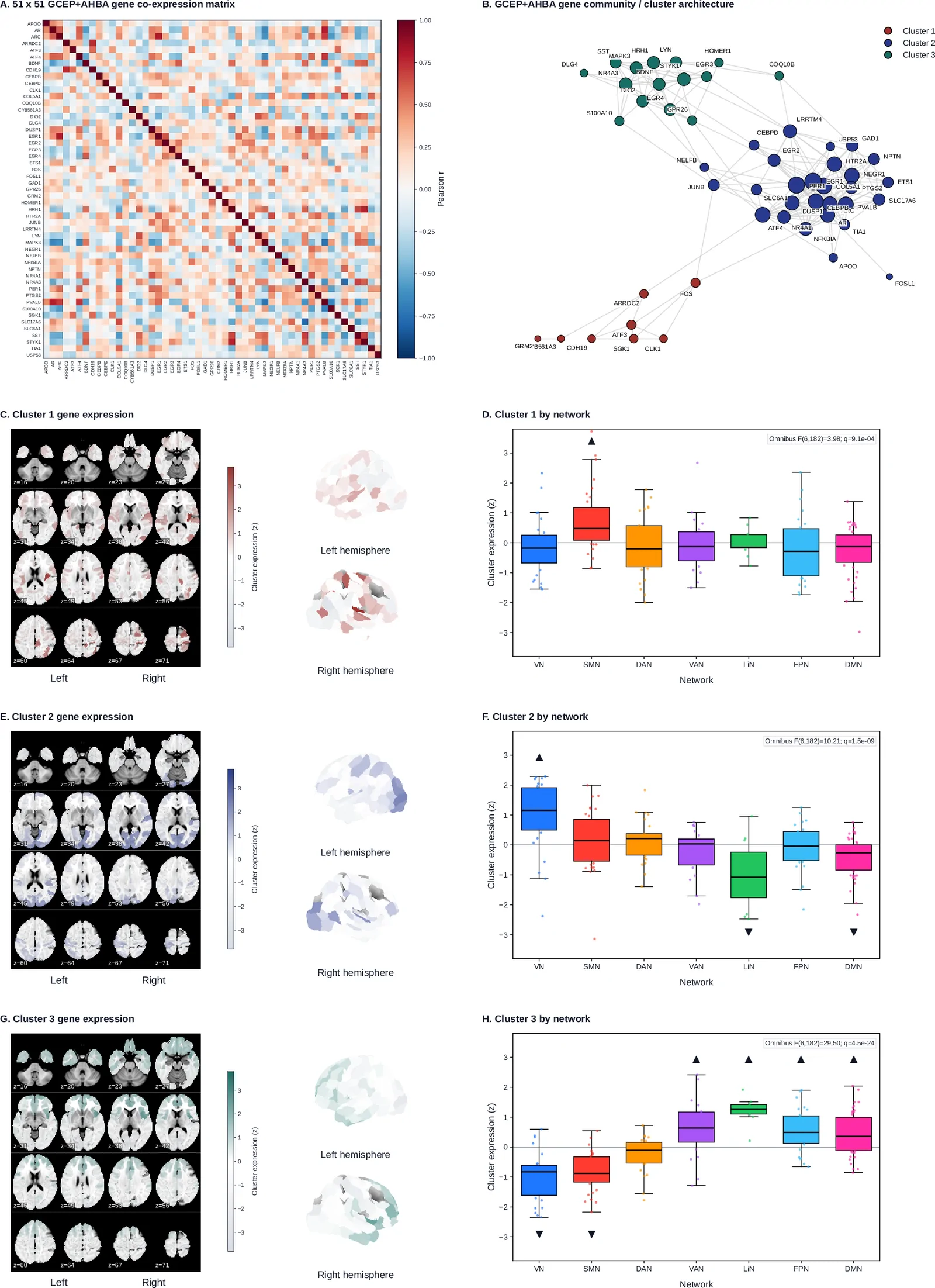
Cortical spatial-expression modules in the retained acute psychedelic-responsive AHBA-overlap set. Panels A-B show the retained GCEP + AHBA co-expression structure (n = 51): the 51 × 51 gene-by-gene spatial co-expression matrix across Schaefer-200 cortical parcels (**A**) and the thresholded positive co-expression graph (Pearson *r* ≥ 0.3), with node size reflecting binary degree and node color reflecting Louvain community assignment (**B**). Panels C-H show average cortical expression maps for Clusters 1–3 after within-cluster *z*-scoring (**C**,**E**,**G**) and corresponding canonical-network parcel distributions (**D**,**F**,**H**). Horizontal lines mark each cluster mean; annotations report BH-corrected omnibus ANOVA results; and triangles mark networks significantly above (▲) or below (▼) zero after BH correction across all 21 cluster-network tests. AHBA: Allen Human Brain Atlas; DAN: dorsal attention network; DMN: default mode network; FPN: frontoparietal network; LiN: limbic network; SMN: somatomotor network; VAN: ventral attention network; VN: visual network.

Correlating Schaefer-200 cortical expression profiles across the GCEP + AHBA set produced a 51 × 51 spatial co-expression matrix, and Louvain community detection on the thresholded *r* ≥ 0.3 graph recovered three cortical spatial-expression clusters (*Q* = 0.691) (Fig. 4A, B).

Cluster 1 (n = 8 genes) showed the broadest cortical distribution and weakest network differentiation, with SMN as the only network centered above the cluster mean (mean *z* = 0.73; BH-adjusted *p* = 1.6 × 10^−3^). The omnibus network test was significant but with the smallest effect size among clusters (*F*(6,182) = 3.98, BH-adjusted *p* = 9.1 × 10^−4^, η2 = 0.12), and post hoc contrasts were driven by higher SMN expression relative to each other network (BH-adjusted *p* ≤ 0.03). *FOS* and *ATF3* were the highest-degree genes in Cluster 1 (degree = 5 each).

Cluster 2 (n = 28 genes) showed a sensory-weighted profile, with VN centered above the cluster mean (mean *z* = 0.98; BH-adjusted *p* = 7.2 × 10^−4^) and LiN and DMN centered below it (LiN mean *z* = −0.99, BH-adjusted *p* = 0.02; DMN mean *z* = −0.38, BH-adjusted *p* = 1.6 × 10^−3^). Network differentiation was significant (*F*(6,182) = 10.21, BH-adjusted *p* = 1.5 × 10^−9^, η2 = 0.25), with VN showing higher mean gene expression than all other canonical networks (BH-adjusted *p* ≤ 0.02). *EGR1* was the highest-degree gene in Cluster 2 (degree = 20), followed by *PVALB* (degree = 16), *ARC* (degree = 15), and *HTR2A* (degree = 15).

Cluster 3 (n = 15 genes) showed the strongest network differentiation and an association/limbic-weighted profile, with LiN, VAN, FPN, and DMN showing gene expression above the cluster mean and VN/SMN below it. The omnibus effect was largest among clusters (*F*(6,182) = 29.50, BH-adjusted *p* = 4.5 × 10^−24^, η2 = 0.49). Post hoc contrasts showed that VAN, LiN, FPN, and DMN showed higher mean gene expression than VN, SMN, and DAN; DAN was higher than VN and SMN; and LiN exceeded VAN, FPN, and DMN (BH-adjusted *p* ≤ 0.03). *NR4A3* and *STYK1* were the highest-degree genes in Cluster 3 (degree = 11 each), with *BDNF* also among the most connected members (degree = 10). Thus, the results define a broad/weakly differentiated Cluster 1, a sensory-weighted Cluster 2, and an association/limbic-weighted Cluster 3.

### HAR overlap and cortical-expansion

**Fig. 5:**
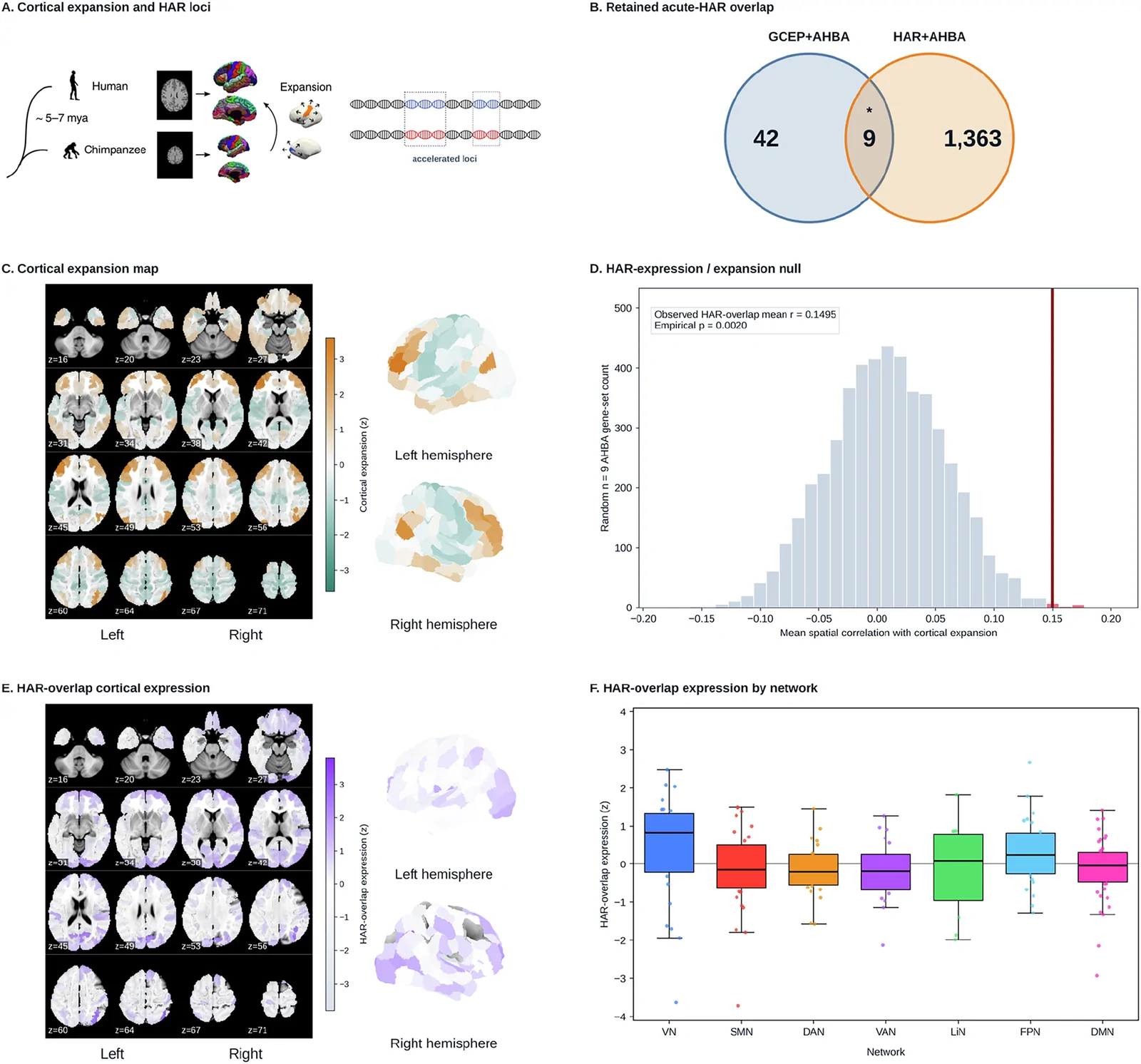
HAR-overlap genes, cortical expansion, and retained acute psychedelic-responsive expression. Panel A provides a schematic linking chimpanzee-to-human cortical expansion with HAR loci (**A**). Panel A was adapted from Fig. 1a, b in Wei et al. [30], © The Author(s) 2019, originally published by Springer Nature in Nature Communications under the Creative Commons Attribution 4.0 International License (https://creativecommons.org/licenses/by/4.0/); changes were made to the layout, labels, and graphical presentation. Panel B depicts an exclusive-count overlap between GCEP + AHBA and HAR + AHBA genes (retained-set-only n = 42; HAR + AHBA-only n = 1,363; overlap n = 9), with the asterisk marking greater-than-chance overlap by one-tailed Fisher exact test (*p* = 0.03) (**B**). Panels C-F compare cortical expansion with the nine-gene HAR + GCEP + AHBA subset: *z*-scored Schaefer-200 cortical-expansion map (**C**), same-size AHBA null test of mean spatial correlation between HAR-overlap expression and cortical expansion using 5000 random gene sets (**D**), *z*-scored mean HAR-overlap expression map (**E**), and canonical-network summary for the same *z*-scored expression values (**F**). AHBA: Allen Human Brain Atlas; DAN: dorsal attention network; DMN: default mode network; FPN: frontoparietal network; GCEP: genes reported to change expression acutely under psychedelics; HAR: human accelerated region; LiN: limbic network; SMN: somatomotor network; VAN: ventral attention network; VN: visual network.

We next asked whether the GCEP + AHBA set intersected with HAR-associated AHBA-overlapping genes (Fig. 5B). Nine retained genes overlapped HAR + AHBA, exceeding chance expectations (one-tailed Fisher exact *p* = 0.03): *CDH19, ETS1, GAD1, GPR26, HTR2A, LRRTM4, NEGR1, SLC17A6*, and *SST*. Their mean cortical expression remained broadly distributed, with no significant canonical-network separation (Fig. 5E, F; conventional parcel-level omnibus ANOVA *p* = 0.17).

The 9-gene subset also showed greater spatial similarity with the cortical-expansion map than same-size AHBA gene sets (mean gene-wise *r* = 0.15, empirical *p* < 0.01; 5000 permutations; Fig. 5C, D; see also Supplementary Figure S4, for absolute correlations).

### Psychostimulant comparator context

As secondary analyses, we compared the GCEP + AHBA set with one acute cortical cocaine comparator and one acute dorsal-striatal amphetamine comparator selected outside the primary psychedelic review (Supplementary Appendix S1).

For cocaine, the comparator was the acute prefrontal-cortex differential-expression source from Emerson et al. [60], corresponding to the PFC Saline-Cocaine vs Saline-Saline comparison. After Ensembl-to-symbol mapping, 343 of 352 genes from the nominal *p* < 0.05 list and 52 of 56 from the *p* < 0.01 sensitivity list were usable for direct comparison. Under both cocaine views, overlap with GCEP + AHBA was zero (one-tailed Fisher exact *p* = 1).

For amphetamine, we evaluated the day-1 dorsal-striatal d-amphetamine source from Crittenden et al. [61], using a bounded 44-gene acute list, with broader 61-gene and 62-gene support views retained as sensitivity analyses. Overlap with GCEP + AHBA was stable across amphetamine-associated gene lists at three genes: *NFKBIA*, *PVALB*, and *S100A10* (one-tailed Fisher exact *p* < 0.005).

### Exploratory psychedelic functional-topography comparison

**Fig. 6:**
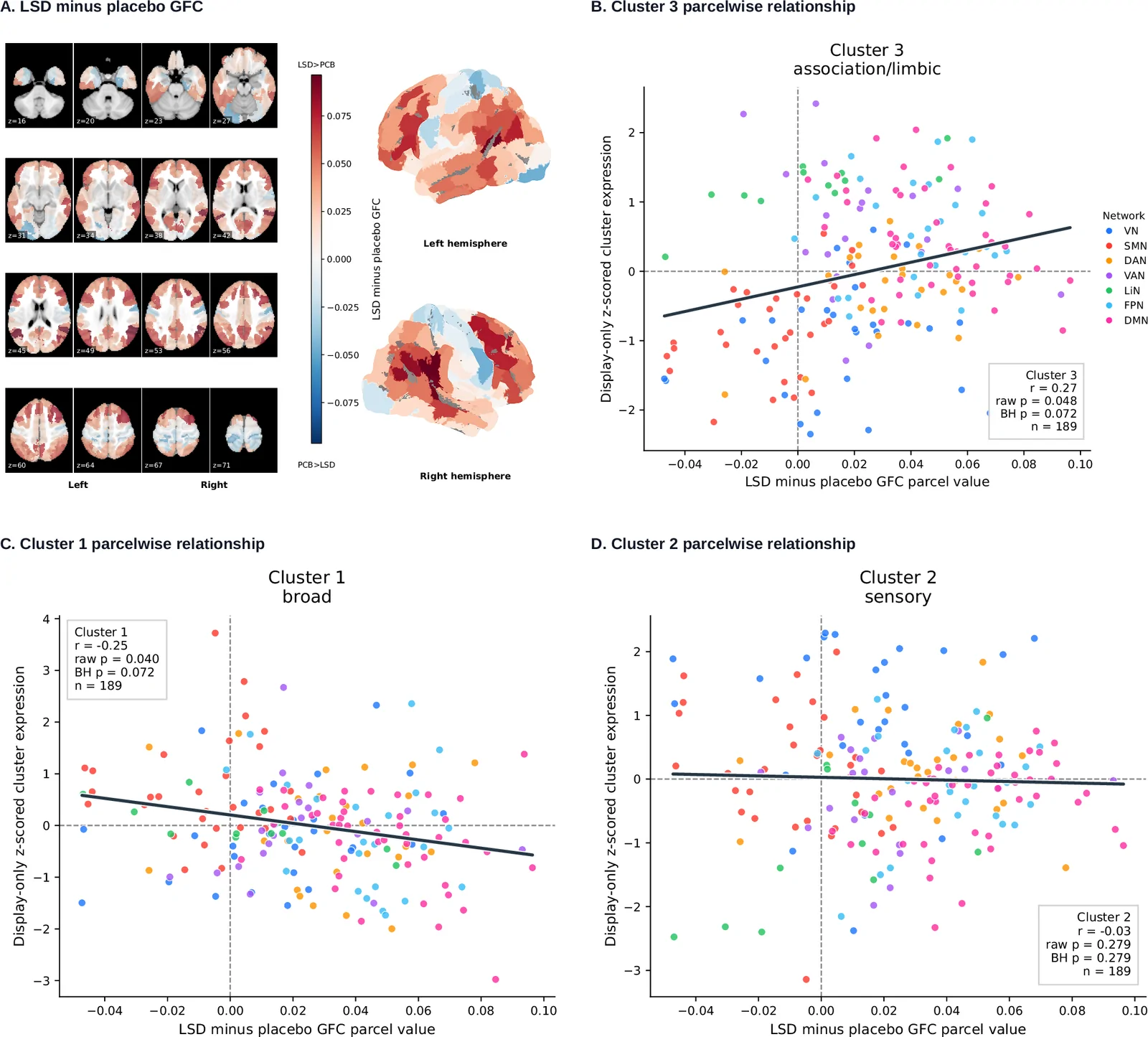
Exploratory cross-modal comparison between LSD-minus-placebo global functional connectivity and Fig. 4, cortical gene-expression clusters. Panel **A** shows the LSD-minus-placebo global functional connectivity (GFC) map in Schaefer-200 cortical space; warmer colors indicate greater GFC on LSD relative to placebo, and cooler colors indicate lower GFC. Panels **B**-**D** show exploratory parcelwise relationships between the LSD-minus-placebo GFC vector and Cluster 3, Cluster 1, and Cluster 2 cortical expression maps across complete Schaefer-200 parcels. Y-axis values show display-only *z*-scored cluster expression. No cluster-versus-GFC test survived Benjamini-Hochberg correction; reported *p*-values are empirical BrainSMASH spatial-null *p*-values rather than ordinary correlation-test *p*-values.

To explore the biological interpretation of identified gene clusters, we assessed the spatial similarity between the gene cluster maps and functional brain changes induced by LSD when compared to placebo. We leveraged resting-state fMRI data from a published study to derive regional estimates of GFC–a measure reflecting how strongly the activity of each brain region is correlated to the activity of all other brain regions. We then assessed GFC differences between the LSD and placebo conditions, revealing GFC increases in prefrontal, medial, and lateral parietal areas under LSD, while GFC decreases on LSD were more prominent among the visual cortices (Fig. 6A). The three clusters differed in exploratory relationships to the LSD-minus-placebo GFC map, but none survived BH correction (Cluster 1 BH-adjusted empirical *p* = 0.07; Cluster 2 *p* = 0.28; Cluster 3 *p* = 0.07). Raw directional correlations were positive for Cluster 3 (*r* = 0.27, raw empirical *p* = 0.05), negative for Cluster 1 (*r* = −0.25, raw empirical *p* = 0.04), and absent for Cluster 2 (*r* = −0.03, raw empirical *p* = 0.28) (Fig. 6B–D).

## Discussion

### Evidence base and acute-set definition

This study converts a heterogeneous preclinical literature into a defined acute gene set for human cortical mapping. The 51 GCEP + AHBA gene set is a descriptive cross-study synthesis, not a uniform transcriptomic signature across drugs, regions, species, or sexes; source studies varied in compounds, assays, neural contexts, timing, and reported directionality. The retained set is therefore best read as an activity-dependent early-response context enriched for immediate-early/activity-regulated transcription, not as a drug-invariant plasticity program.

### Gene enrichment and ontology of the retained acute set

CSEA, complementary SEA, and ontology results converged on a cortex-centered, rather than cortex-exclusive, interpretation of the GCEP + AHBA set. The ontology signal pointed toward neuronal projection and synaptic organization, based on Enrichr and SynGO libraries [55, 56]. The Dougherty CSEA/SEA labels should be read as a mouse CNS reference-enrichment context rather than direct evidence of cell-type-resolved recruitment in humans [57], especially because the downstream human maps are derived from bulk AHBA/imaging-transcriptomic workflows [51, 52, 54].

The narrower cortical rows are therefore best interpreted as a bounded projection-neuron reference signal, not as evidence of direct laminar or cell-class recruitment. *GRM2* links this enrichment surface to serotonin-glutamate signaling and alcohol-seeking-control context [46, 64, 65]. *EGR4* contributes by adding a neuronal transcriptional regulation context, including *BDNF/KCC2*-linked evidence, but does not by itself establish projection-neuron specificity [66, 67]. *LRRTM4* further supports an excitatory synapse organization framework [68].

Taken together, Fig. 2 supports a cortex-centered, activity-linked projection/synaptic context that is consistent with prior evidence that psychedelics can elicit complex transcriptional responses and plasticity-related cortical programs [16, 18–21, 43, 48, 49]. This interpretation is also compatible with emerging human diffusion evidence reporting microstructural changes after first-time psilocybin use in prefrontal-subcortical tracts, including PFC-striatum and PFC-thalamus tracts [69]. That bridge remains contextual, and these marker-level contributors do not justify pathway, cell-type, laminar, circuit, or translational mechanism claims.

### Serotonergic spatial alignment in human cortex

The above-chance alignment between GCEP + AHBA (excluding *HTR2A*) and the cortical *HTR2A* expression map places the animal-model transcriptomic findings in a human cortical-expression framework directly relevant to 5-HT2A receptor-centered models of classical psychedelic action [7, 25, 27–29]. The broader marker panel sharpened rather than weakened this interpretation: SLC6A4, encoding the serotonin transporter (SERT), showed a similar mean alignment to *HTR2A*, and both exceeded *HTR2C* and *DRD1*. This pattern is consistent with prior in vivo evidence for regional relationships between SERT and 5-HT2A receptor binding [70] and supports an *HTR2A/SLC6A4*-centered serotonergic cortical-expression pattern rather than a nonspecific serotonergic receptor or dopaminergic receptor alignment effect. Because these maps are derived from AHBA transcriptomic data, the results reflect correlative spatial alignment rather than direct measures of receptor density or receptor-specific regulation.

### Cortical spatial-expression modules

Cortical co-expression clustering separated the GCEP + AHBA genes into three spatial-expression modules that are best interpreted as complementary molecular profiles, rather than separable pathways or validated circuit mechanisms.

Cluster 1 was broadly distributed and only weakly differentiated across canonical networks, with SMN as the only network showing above-mean gene expression. Its broad acute-response interpretation is anchored by high-degree *FOS* and *ATF3* in the graph and by source studies reporting acute psychedelic-related induction of immediate-early or plasticity-associated transcriptional programs [21, 48, 50]. *GRM2* contributes the most relevant translational seed within this cluster because mGlu2-related prefrontal/cortical signaling has been linked to 5-HT2A/glutamate interactions and alcohol-seeking control in preclinical work, but this should be treated as hypothesis-generating context rather than evidence that the module indexes relapse circuitry [64, 65, 71–73].

Cluster 2 showed a sensory-weighted profile, centered above the cluster mean in VN and below it in LiN and DMN. Its strongest interpretation is therefore a sensory-weighted activity-responsive signaling component, including immediate-early/activity-regulated genes [42, 44, 74–78] and linked to the serotonergic system through *HTR2A* [79, 80], without implying a discrete *HTR2A* program, sensory-perceptual mechanism, or cell-type-specific recruitment.

Cluster 3 showed the strongest network differentiation and the clearest association/limbic weighting, with LiN, VAN, FPN, and DMN above the cluster mean and VN/SMN below it. This module provides the main plasticity and synaptic-organization context for the retained set, with *BDNF, HOMER1, DLG4/PSD95*, and *EGR4* supporting a cautious trophic, postsynaptic, and neuronal-transcriptional reading [66, 67, 81–85]. *SST* adds inhibitory somatostatin-interneuron context relevant to addiction-related stress and alcohol-use microcircuit models in prefrontal/striatal systems [86–89]. Its association/limbic spatial profile is compatible with broader psychedelic work on structural remodeling and transmodal or affective-network modulation [2, 18, 90–94], but it does not establish a receptor- or circuit-specific mechanism.

Across the retained set, the three-cluster solution reflects a compact organizing scheme rather than a validated pathway taxonomy. Together with prior imaging-transcriptomic and pharmacological neuroimaging work [22–24, 95], it nominates perturbational hypotheses, including addiction-relevant prefrontal/projection hypotheses raised by *GRM2, EGR4*, and *SST* [65–67, 71–73, 86–89], while preserving the central limitation that AHBA spatial co-expression cannot identify causal pathways, network mechanisms, or clinical phenotypes.

### HAR overlap and cortical-expansion context

The HAR analysis suggests that the GCEP + AHBA set is modestly overrepresented among HAR-associated AHBA-overlapping genes [32], providing an evolutionary-genomic and spatially bounded signal. HARs are frequently linked to neurodevelopmental enhancer biology [32, 96], and human cortical expansion is especially prominent in higher-order association cortex [30, 31, 33]. The HAR-overlap expression map was broadly cortical, did not show significant canonical-network separation, and showed greater-than-random spatial alignment with the cortical-expansion surface.

At a compact gene-context level, the subset retains several interpretable anchors. *HTR2A* provides links to the serotonergic system [7]. *LRRTM4* and *NEGR1* support synaptic-organization and synaptic-targeting context, with *GAD1* extending this into inhibitory GABA biology [68, 97, 98]. *SST* adds a corticolimbic/frontostriatal microcircuit signal relevant to stress and alcohol-use models [86–89].

Accordingly, the HAR analysis narrows the hypothesis space for future cortical-evolution and circuit-centered tests, including addiction- and affective-circuit hypotheses, but does not resolve gene-specific mechanisms or establish a causal link between psychedelic pharmacology and human cortical evolution.

### Psychostimulant comparator context

The psychostimulant comparator analyses argue against the simplest nonspecific alternative, namely that the retained GCEP + AHBA signal merely reflects a generic acute psychostimulant response. The acute cortical cocaine comparator showed no direct overlap with the retained 51-gene set, whereas the best-effort amphetamine comparator showed only a small, although significant, stable 3-gene overlap [60, 61]. These analyses remain insufficient to establish psychedelic specificity, but they provide bounded contextual evidence against the simplest generic acute-psychostimulant explanation.

### Exploratory psychedelic functional-topography context

The functional-topography comparison provides a cross-modal framework, not evidence that AHBA modules explain LSD-related connectivity. Because no cluster-versus-GFC test survived BH correction, the raw directions remain visually descriptive and hypothesis-generating only.

### Limitations

Several limitations constrain inference in this study. First, no analyzable human neural transcriptomic gene list met eligibility criteria, so the translational bridge remains indirect. Second, the retained acute gene set is a descriptive cross-study synthesis rather than a uniform signature across drugs, regions, species, or sexes, and these analyses were exploratory and were not preregistered. Third, the evidence base was strongly male-skewed and temporally sparse beyond the retained acute window; exact 5 h observations added no unique AHBA-overlapping genes beyond the within-3 h set, and later AHBA-overlap observations were limited to a 24 h psilocybin pig study [63] and an LSD-only >24 h mouse lineage. Fourth, publication bias and preferential reporting of positive findings remain plausible in a literature dominated by targeted candidate-gene studies and incompletely recoverable transcriptome-wide reports. Fifth, the human mapping relied on bulk AHBA microarray data from only six adult postmortem donors and was restricted downstream to Schaefer-200 cortical parcel space, making the spatial analyses correlative by design, excluding subcortical structures from joint analysis, and precluding cell-type-resolved mechanisms. Finally, the *HTR2A* alignment, cortical modules, HAR overlap/cortical-expansion alignment, psychostimulant comparator findings, and functional-topography findings should be read as bounded spatial or contextual correspondences rather than as evidence of receptor-specific regulation, causal circuit mechanisms, psychedelic specificity, or evolutionary explanations of psychedelic action.

### Implications for translational psychiatry and future directions

Despite these limitations, this study provides a bounded translational framework for mapping preclinical psychedelic-responsive gene sets onto human cortical expression, receptor-marker, cell-enrichment, and cortical-expansion. The findings should be read as convergent spatial and enrichment correspondences that nominate testable hypotheses for future experimental and clinical studies, rather than as evidence that the retained genes causally shape human brain function or structure.

Given ongoing clinical interest in psychedelic-assisted treatment for alcohol-use disorder and the addiction-relevant prefrontal, corticolimbic, and frontostriatal context raised by the present analyses, future work should test these hypotheses in disorder-specific, cell-resolved, and circuit-informed frameworks [13, 65–67, 71, 72, 73, 86–89].

Future work should prioritize analyzable human neural transcriptomics, sex-balanced acute designs, denser later-window sampling, joint cortical-subcortical mapping, and cell-resolved follow-up, especially given cell-class- and stage-specific human cortical maturation [99].

## Supplementary information

Supplementary Information (Unified Supplement)

Supplementary Table S1: Record-level Screening and Eligibility Ledger

Supplementary Table S3: Study-level Provenance Summary

Supplementary Table S4: Scoped-corpus Evidence-hierarchy Accounting

Supplementary Data 1: Original Extraction Log

## Data Availability

Curated screening records, extracted gene and study metadata, and the scoped-corpus accounting surfaces supporting the literature review are available in Supplementary Tables S1, S3, and S4 and Supplementary Appendix S1. The original extraction log is provided in the supplement source-data bundle as Original_Extraction_Log. The retained acute gene sets, AHBA-overlap tables, and the parcelized derivative surfaces supporting the reported cortical analyses are available from the corresponding author on reasonable request. The LSD-minus-placebo GFC surface used for Fig. 6 was derived from the published dataset of Carhart-Harris et al. [1].

## Code availability

The versioned analysis and figure-generation scripts used for this study can be provided by the first or corresponding author upon reasonable request.
