# Supplementary material for: Cross-species mapping of acute psychedelic-responsive genes links HTR2A cortical expression, cell-type enrichment, and human cortical expression modules
Source: Transl Psychiatry. 2026 Aug 1;16:594. doi: 10.1038/s41398-026-04349-z (PMC13720183; doi:10.1038/s41398-026-04349-z)
Supplement: Supplementary file 1 [file 41398_2026_4349_MOESM1_ESM.docx]

**Supplementary Materials for:**

Cross-species mapping of acute psychedelic-responsive genes links HTR2A cortical expression, cell-type enrichment, and human cortical expression modules

**Authors:**

Patrick A. McConnell^2^, Jackson Raffety^1^, Andrew Li^1^, Eric Steinberg^1^, Syed Rahim^1^, Christian Valtierra^1^, Adam Gazzaley^1^, Robin Carhart-Harris^1^, Lorenzo Pasquini^1, 2,^ *

**Total Content:**

- Supplementary Methods: 1
- Supplementary Figures: 4
- Supplementary Tables: 8
- Supplementary Appendices: 2
- Supplementary Data: 1

**Internal Unified Content:**

- Supplementary Methods
- Supplementary Figures S1-S4
- Supplementary Tables S2, S5-S8
- Supplementary Appendices S1-S2

**Non-Unified Content:**

- Supplementary_Material_v5.0.1_Table_S1.csv
- Supplementary_Material_v5.0.1_Table_S3.csv
- Supplementary_Material_v5.0.1_Table_S4.csv
- Supplementary_Data_1_Original_Extraction_Log.xlsx

**Supplementary Methods**

**Neuroimaging data acquisition and preprocessing**. The LSD functional MRI data used in the present study were derived from a previously published and publicly available dataset of Carhart-Harris et al. In that study, healthy participants underwent resting-state fMRI under placebo and LSD in a double-blind, within-subject design. Structural and functional MRI data were acquired on a 3T GE HDx system. High-resolution anatomical images were obtained using a 3D fast spoiled gradient echo sequence (1 mm isotropic resolution). Resting-state BOLD fMRI data were acquired using a gradient-echo echo-planar imaging sequence (TR = 2000 ms, TE = 35 ms, 35 axial slices, 3.4 mm isotropic voxels). Two resting-state scans were collected per session, each lasting approximately 7.3 minutes. Functional MRI data preprocessing was conducted using a combination of established neuroimaging software packages, including FSL, AFNI, FreeSurfer, and ANTs. One participant discontinued scanning due to anxiety, and four additional participants were excluded because of excessive head motion. Motion was quantified using framewise displacement (FD), with exclusion criteria based on the proportion of scrubbed volumes exceeding predefined thresholds. Following exclusions, fifteen participants were retained for analysis. Preprocessing steps included removal of initial volumes, despiking, slice-timing correction, motion correction, brain extraction, registration to individual anatomical images, and nonlinear normalization to MNI space. Volumes exceeding FD thresholds were scrubbed and replaced with the mean of adjacent volumes. Additional steps included spatial smoothing (6 mm FWHM), band-pass filtering (0.01–0.08 Hz), detrending, and regression of nuisance signals. Nuisance regressors included six motion parameters and three anatomically derived signals (ventricles, draining veins, and local white matter). Local white matter signals were estimated using a voxelwise neighborhood approach to reduce non-neural noise. The Schaefer 200 parcel atlas was then used to derive time series estimates for each brain region and global functional connectivity maps were derived as described in the main body of the manuscript. No additional statistical inference was performed within the LSD dataset beyond spatial correlation analyses.

**Supplementary Figure S1**

**Supplementary Figure S1. Original Covidence screening flow.** We retained this image as the authoritative visual record of the database screening stages. The later record-level screening and eligibility ledger is provided in Supplementary Table S1. The literature search strategy together with the explicit title/abstract and initial full-text screening criteria is retained in Supplementary Appendix S1.

**Supplementary Figure S2**

**Supplementary Figure S2.** AHBA expression heat map across the Schaefer-200 cortical atlas after abagen processing. Rows are cortical parcels and columns are retained genes. Color shows normalized expression from lower to higher values. Pale rows mark parcels with no retained values. The exact list of the 11 all-missing parcels is given in Supplementary Appendix S2 and Supplementary Table S2.

**Supplementary Figure S3**

**Supplementary Figure S3. Time-binned view of the AHBA-overlapping gene set across the reviewed studies.** Each dot marks a gene-by-time-bin combination, and larger dots indicate more unique study-time observations. The single 5 h point is already represented within the <3 h set, so the cumulative AHBA-overlapping gene set is unchanged at <=3 h and <=5 h. The empty >5-12 h row is shown intentionally to make the sampling gap visible.

**Supplementary Figure S4**

**Supplementary Figure S4.** Supportive cortical-expansion null comparison for the nine-gene HAR-overlap subset. This supplement supports the main-text Figure 5D cortical-expansion null rather than presenting a separate inferential analysis. Panel A shows the null distribution for the mean spatial correlation statistic, and panel B shows the corresponding null distribution for the mean absolute correlation statistic, each generated from 5,000 size-matched random AHBA gene sets. The red line marks the observed HAR-overlap subset statistic.

**Supplementary Table S2**

**Supplementary Table S2. Schaefer parcels with all values missing after AHBA processing.** This table lists the 11 right-hemisphere parcels that were entirely missing in expression_schaefer.csv.

Key: 7Networks = Schaefer 7-network atlas naming convention; RH = right hemisphere; Vis = Visual network; SomMot = Somatomotor network; SalVentAttn = Salience/Ventral Attention network; Cont = Frontoparietal Control network; Default = Default Mode network.

| parcel_number | hemisphere | parcel_name | status |
| --- | --- | --- | --- |
| 114 | R | 7Networks_RH_Vis_14 | all_values_missing_in_expression_schaefer |
| 121 | R | 7Networks_RH_SomMot_6 | all_values_missing_in_expression_schaefer |
| 128 | R | 7Networks_RH_SomMot_13 | all_values_missing_in_expression_schaefer |
| 132 | R | 7Networks_RH_SomMot_17 | all_values_missing_in_expression_schaefer |
| 151 | R | 7Networks_RH_SalVentAttn_PrC_1 | all_values_missing_in_expression_schaefer |
| 158 | R | 7Networks_RH_SalVentAttn_Med_3 | all_values_missing_in_expression_schaefer |
| 166 | R | 7Networks_RH_Cont_Par_2 | all_values_missing_in_expression_schaefer |
| 175 | R | 7Networks_RH_Cont_PFCl_6 | all_values_missing_in_expression_schaefer |
| 176 | R | 7Networks_RH_Cont_PFCl_7 | all_values_missing_in_expression_schaefer |
| 177 | R | 7Networks_RH_Cont_pCun_1 | all_values_missing_in_expression_schaefer |
| 193 | R | 7Networks_RH_Default_PFCdPFCm_3 | all_values_missing_in_expression_schaefer |

**Supplementary Table S5**

**Supplementary Table S5.** GCEP+AHBA genes in Clusters 1-3. This staged carry-forward table lists the retained GCEP+AHBA genes grouped by the three cluster assignments used in the module analyses.

| Cluster | n_genes | Genes |
| --- | --- | --- |
| Cluster 1 | 8 | ARRDC2; ATF3; CDH19; CLK1; CYB561A3; FOS; GRM2; SGK1 |
| Cluster 2 | 28 | APOO; AR; ARC; ATF4; CEBPB; CEBPD; COL5A1; DUSP1; EGR1; EGR2; ETS1; FOSL1; GAD1; HTR2A; JUNB; LRRTM4; NEGR1; NELFB; NFKBIA; NPTN; NR4A1; PER1; PTGS2; PVALB; SLC17A6; SLC6A1; TIA1; USP53 |
| Cluster 3 | 15 | BDNF; COQ10B; DIO2; DLG4; EGR3; EGR4; GPR26; HOMER1; HRH1; LYN; MAPK3; NR4A3; S100A10; SST; STYK1 |

**Supplementary Table S6**

**Supplementary Table S6.** Raw numbers of gene lists involved in the analyses and their sources. Note. Time-window counts are not mutually exclusive. Four genes (ARC, DUSP1, FOS, and JUNB) appear in both the <=3 h and >12 h windows. “Brain-expressed genes” = Allen Human Brain Atlas (post-abagen Schaefer-200 cortical expression background; see Supplementary Appendix S2).

| **Gene lists** | **Number of unique genes** | **Source** |
| --- | --- | --- |
| Brain-expressed genes | 15,633 | Allen Human Brain Atlas |
| Pre-AHBA HAR-associated gene list | 2,164 | Doan et al. 2016 |
| Genes changing expression under acute psychedelic administration in animal models (within <=3 h) | 56 | Benekareddy et al. 2012; Desouza et al. 2021; Fadahunsi et al. 2022; Gewirtz et al. 2002; González-Maeso et al. 2003, 2007; Jefsen et al. 2021; Lerer et al. 2024; Martin et al. 2016; Nichols et al. 2002, 2003, 2004; Santini et al. 2014; Tilakaratne et al. 1996; Vaidya et al. 1997 |
| Subacute changes (>12 h) in gene expression after psychedelic administration in animal models | 104 | Donovan et al. 2021; Nardou et al. 2023 |
| Bounded comparator gene list: cocaine (Sal-Coc vs Sal-Sal in PFC) | 343 | Emerson et al. 2023 |
| Bounded comparator gene list: d-amphetamine (day 1 dorsal-striatal source) | 44 | Crittenden et al. 2021 |

**Supplementary Table S7**

**Supplementary Table S7.** List of GCEP+AHBA genes. Genes in bold also overlapped with HAR+AHBA genes.

**List of GCEP+AHBA**

APOO, AR, ARC, ARRDC2, ATF3, ATF4, BDNF, **CDH19**, CEBPB, CEBPD, CLK1, COL5A1, COQ10B, CYB561A3, DIO2, DLG4, DUSP1, EGR1, EGR2, EGR3, EGR4, **ETS1**, FOS, FOSL1, **GAD1**, **GPR26**, GRM2, HOMER1, HRH1, **HTR2A**, JUNB, **LRRTM4**, LYN, MAPK3, **NEGR1**, NELFB, NFKBIA, NPTN, NR4A1, NR4A3, PER1, PTGS2, PVALB, S100A10, SGK1, **SLC17A6**, SLC6A1, **SST**, STYK1, TIA1, USP53

**Supplementary Table S8**

**Supplementary Table S8.** Acute GCEP genes not present in the AHBA background.

**List of acute GCEP not present in the AHBA**

GLUT4, N10, IL6, ILAD1, NGFLC

**Supplementary Appendix S1**

This appendix retains the literature search strategy together with the explicit title/abstract and initial full-text screening criteria.

The database screening flow is shown in Supplementary Figure S1, the record-level screening ledger is provided in Supplementary Table S1, and the scoped-corpus evidence-hierarchy accounting surface is provided in Supplementary Table S4.

The original extraction log is retained separately as Supplementary Data 1 for transparency.

**Explicit PICO-S screening criteria**

**Population**

**Include**

- All humans and other animals are eligible.
- Neuronal cell cultures or other biological tissue samples (e.g., plasma) from which to assess genetic and epigenetic changes post-psychedelic administration are eligible.

**Exclude**

- Plants, fungi, insects, artificial intelligence, computational models of brain function, simulations
- Non-neuronal studies, peripheral tissues without a clear link to brain or neuronal activity.

**Intervention / Exposure**

**Include**

- Trials administering a 5-HT2A receptor agonist classical psychedelic such as LSD, N,N-DMT, ayahuasca, mescaline, DOI, or psilocybin on 1-2 study visits are eligible.
- At least one trial group must receive a 5-HT2A receptor agonist classical psychedelic with acute primary neurogenetic outcomes assessed for the study to be eligible.

**Exclude**

- Data from trials focusing on genetic expression impact of atypical psychedelics such as 5-MeO-DMT, S-/ R- Ketamine, Ibogaine, Salvia, MDMA, cannabis, or THC, or other will be excluded, unless used as a control against one of the permitted classical psychedelic compound's action.

**Comparator / Context**

**Include**

- Eligible drug administration
- Eligible control or baseline control implemented
- Acute primary neurogenetic outcomes assessed relative to the control condition.

**Exclude**

- No eligible drug administration
- No acute primary neurogenetic outcomes
- No form of pre/post, vehicle, or placebo control.

**Outcome**

**Include**

- 1 - Data dictionary of acute gene expression profile changes after classical psychedelic administration.
- 1 - Data dictionary of brain regions and brain cell-type specific targets most enriched for the most impacted genes.
- 2 - Exploratory analyses of sub-acute and chronic changes observed, if any.

**Exclude**

- No reporting on genetic markers related to neural function acutely post-psychedelic experience.

**Study Characteristics**

**Include**

- Placebo-controlled, Baseline-controlled (Pre-/Post), and/or Cross-Sectional drug administration studies are all eligible.
- The acute effects of psychedelic action must be assessed (within same day maximum), but additional instances of sub-acute and long-term/chronic datasets may be considered as secondary or exploratory outcomes.

**Exclude**

- case studies, review articles, non-English articles, opinion pieces, observational studies, qualitative reports, preprints, conference abstracts, theses and dissertations, secondary analyses, and non-peer reviewed publications will not be eligible.

**Search strings used for PubMed and Google Scholar**

**Query 1**

("hallucinogens" OR psychedelic OR psilocybin OR "lysergic acid diethylamide" OR LSD OR DMT OR dimethyltryptamine OR "5-HT2A agonist" OR "serotonin 2A receptor agonist") AND ("gene expression" OR "gene expression profiling" OR transcriptome OR transcriptomics OR "RNA sequencing" OR "differential gene expression") AND ("brain" OR "brain tissue" OR "cerebral cortex" OR hippocampus OR "prefrontal cortex") AND (acute OR "single dose" OR "short-term")

**Query 2**

("gene expression" OR transcriptome OR transcriptomics) AND ("hallucinogen" OR psychedelic) AND ("psilocybin" OR "lysergic acid diethylamide" OR "LSD" OR "DMT" OR "dimethyltryptamine" OR "mescaline" OR "DOI" OR "ayahuasca") AND ("brain" OR neural OR "neuron" OR "cortical" OR "cortex" OR "cell culture")

**Query 3**

(gene expression) AND (5-HT2A) AND (psychedelic OR hallucinogen)

**Secondary bounded psychostimulant comparator adjudication**

This secondary comparator adjudication was conducted outside the main PRISMA-informed psychedelic scoping screen and was used only to provide a limited nonspecific-psychostimulant context for the retained acute psychedelic-responsive set. Candidate comparator sources were considered only if they provided an explicit acute gene-level neural-expression contrast with a recoverable gene-list surface suitable for symbol-level comparison. Because the main manuscript analyses are cortex-centered, cortical tissue matching was prioritized when available; where tissue matching was not available, that limitation was retained explicitly rather than treated as equivalent.

For cocaine, the retained acute comparator was the prefrontal cortex contrast reported by Emerson et al. 2023 (62), corresponding to the acute Sal-Coc versus Sal-Sal differential-expression surface for first-time cocaine exposure in PFC tissue. For amphetamine, the retained acute comparator was the day-1 dorsal-striatal d-amphetamine source reported by Crittenden et al. 2021 (61), reflecting tissue harvested 20 min after the first injection. Because the amphetamine source describes a filtered acute 44-gene subset while the directly recoverable displayed and day-1 source surfaces are larger, the manuscript’s amphetamine comparator was treated as a bounded best-effort reconstructed acute list rather than as a perfectly recovered author-supplied table, and the dorsal-striatal tissue limitation was kept explicit.

These comparator sources were used only for bounded overlap and contextual enrichment checks. They were not used to define, filter, or validate the primary psychedelic gene set, and they were not treated as matched positive or negative controls.

**Supplementary Appendix S2**

This appendix records the AHBA processing steps used to build the cortical expression matrix.

**Abagen Code:**

$ abagen --output-file Schaeffer_200.csv Schaeffer_200.nii.gz

**Abagen Report:**

Regional microarray expression data were obtained from 6 post-mortem brains (1 female, ages 24.0--57.0, 42.50 +/- 13.38) provided by the Allen Human Brain Atlas (AHBA, https://human.brain-map.org; [H2012N]). Data were processed with the abagen toolbox (version 0.1.3; <https://github.com/rmarkello/abagen>) using a 200-region volumetric atlas in MNI space.

First, microarray probes were reannotated using data provided by [A2019N]; probes not matched to a valid Entrez ID were discarded. Next, probes were filtered based on their expression intensity relative to background noise [Q2002N], such that probes with intensity less than the background in >=50.00% of samples across donors were discarded, yielding 31,569 probes. When multiple probes indexed the expression of the same gene, we selected and used the probe with the most consistent pattern of regional variation across donors (i.e., differential stability; [H2015N]), calculated with:

$$\Delta S\left( p \right)= \frac{1}{\left( \begin{matrix} N \\ 2 \end{matrix} \right)} \sum_{i=1}^{N-1} \sum_{j=i+1}^{N} \rho\left[ B_{i}\left( p \right), \right]B_{j}\left( p \right)$$

Where $\rho$ is Spearman's rank correlation of the expression of a single probe, p, across regions in two donors B_i_ and B_j_ and N is the total number of donors. Here, regions correspond to the structural designations provided in the ontology from the AHBA

The MNI coordinates of tissue samples were updated to those generated via non-linear registration using the Advanced Normalization Tools (ANTs; <https://github.com/chrisfilo/alleninf>). Samples were assigned to brain regions in the provided atlas if their MNI coordinates were within 2 mm of a given parcel. All tissue samples not assigned to a brain region in the provided atlas were discarded.

Inter-subject variation was addressed by normalizing tissue sample expression values across genes using a robust sigmoid function [F2013J]:

$$\Delta S\left( p \right)= \frac{1}{\left( \begin{matrix} N \\ 2 \end{matrix} \right)} \sum_{i=1}^{N-1} \sum_{j=i+1}^{N} \rho\left[ B_{i}\left( p \right), \right]B_{j}\left( p \right)$$

where $\left\langle x \right\rangle$ is the median and ${IQR}_{z}$is the normalized interquartile range of the expression of a single tissue sample across genes. Normalized expression values were then rescaled to the unit interval:

$$x_{scaled}= \frac{x_{norm}-min\left( x_{norm} \right)}{max\left( x_{norm} \right)-min\left( x_{norm} \right)}$$

Gene expression values were then normalized across tissue samples using an identical procedure. Samples assigned to the same brain region were averaged separately for each donor and then across donors, yielding a regional expression matrix with 200 rows, corresponding to brain regions, and 15,633 columns, corresponding to the retained genes.

**REFERENCES:**

[A2019N]: Arnatkevic̆iūtė, A., Fulcher, B. D., & Fornito, A. (2019). A practical guide to linking brain-wide gene expression and neuroimaging data. Neuroimage, 189, 353-367.

[F2013J]: Fulcher, B. D., Little, M. A., & Jones, N. S. (2013). Highly comparative time-series analysis: the empirical structure of time series and their methods. Journal of the Royal Society Interface, 10(83), 20130048.

[H2012N]: Hawrylycz, M. J., Lein, E. S., Guillozet-Bongaarts, A. L., Shen, E. H., Ng, L., Miller, J. A., ... & Jones, A. R. (2012). An anatomically comprehensive atlas of the adult human brain transcriptome. Nature, 489(7416), 391-399.

[H2015N]: Hawrylycz, M., Miller, J. A., Menon, V., Feng, D., Dolbeare, T., Guillozet-Bongaarts, A. L., ... & Lein, E. (2015). Canonical genetic signatures of the adult human brain. Nature Neuroscience, 18(12), 1832.

[Q2002N]: Quackenbush, J. (2002). Microarray data normalization and transformation. Nature Genetics, 32(4), 496-501.
